# Effect of intraoperative pressure ulcer preventive nursing on inflammatory markers in patients with high-risk pressure ulcers: A protocol of systematic review and meta-analysis

**DOI:** 10.1097/MD.0000000000020254

**Published:** 2020-05-15

**Authors:** Feng Jin, Yan-jun Fu, Yang Zhang, Jing-ling Yan, Kui-chen Zhou, Hong-wei Liu

**Affiliations:** a Department of Surgery; b Department of Laboratory; c Department of Ophthalmology, First Affiliated Hospital of Jiamusi University, Jiamusi, China.

**Keywords:** effects, inflammatory markers, pressure ulcer, preventive nursing

## Abstract

**Background:**

This study will be designed to appraise the effects of intraoperative pressure ulcer preventive nursing (IPUPN) on inflammatory markers (IMs) in patients with high-risk pressure ulcers (HRPU) based on high quality randomized controlled trials (RCTs).

**Methods:**

In this study, we will perform a rigorous literature search from the following electronic databases: Cochrane Library, MEDLINE, Embase, Cumulative Index to Nursing and Allied Health Literature, Allied and Complementary Medicine Database, and Chinese Biomedical Literature Database. All electronic databases will be retrieved from their initial time to March 1, 2020 without limitations of language and publication status. We will only consider high quality RCTs that explored the effects of IPUPN on IMs in patients with HRPU. Two investigators will identify relevant trials, extract data, and appraise risk of bias in each eligible trial. Data will be pooled by either a fixed-effects model or a random-effects model according to the results of heterogeneity identification. The primary outcomes include IMs, and incidence of new pressure ulcers. The secondary outcomes are time to ulcer development, quality of life, length of hospital stay, and adverse events. Statistical analysis will be undertaken using RevMan 5.3 software.

**Results:**

This study will summarize high quality clinical evidence of RCTs to evaluate the effects of IPUPN on IMs in patients with HRPU.

**Conclusion:**

The expected findings may provide helpful evidence to determine whether IPUPN is an effective intervention on IMs in patients with HRPU.

**INPLASY Registration Number:**

INPLASY202040029.

## 1 Introduction

Pressure ulcers (PUs) are very common public health issue.^[1,2]^ They occur when the skin is exposed to pressure and shear,^[3,4,5]^ especially in patients under conditions of long-term mobility or immobility, poor nutrition status, compromised blood flow, and neuropathy sensation.^[6,7,8,9]^ Published study has reported that the prevalence of PUs ranges from 0.3% to 46%, and its incidence varies between 0.8% and 34%.^[10]^ Thus, it is very important to prevent PUs in patients with high-risk PUs (HRPUs),^[6,11,12,13]^ which is associated with inflammatory markers (IMs).^[14,15,16,17]^ Fortunately, intraoperative PU preventive nursing (IPUPN) has found to be effectively preventing PUs and impacting IMs in patients with HRPU.^[18,19,20,21,22,23,24,25,26]^ However, no study has investigated the effects of IPUPN on IMs in patients with HRPU. Therefore, this study will systematically and comprehensively explore the effects of IPUPN on IMs in patients with HRPU.

## 2 Methods

### 2.1 PROSPERO registration

This protocol has been reported based on the Preferred Reporting Items for Systematic Reviews and Meta-Analysis Protocol (PRISMA-P) statement guidelines,^[27]^ and it has been registered in the INPLASY202040029.

### 2.2 Ethics and dissemination

No ethical approval is required in this study, because it will only analyze published data. It is supposed to be published on a peer-reviewed journal or presented in a conference meeting.

### 2.3 Inclusion criteria for study selection

#### 2.3.1 Type of studies

This study will only include high quality randomized controlled trials (RCTs) that assessed the effects of IPUPN on IMs in patients with HRPU. We will exclude other types of studies, such as non-RCTs, quasi-RCTs, case studies, reviews, and animal studies.

#### 2.3.2 Type of participants

Any participants (18 years old or over) who have been diagnosed as HRPU and received IPUPN will be included in spite of race, nationality, and sex.

#### 2.3.3 Type of interventions

All patients in the experimental group were treated with any types of IPUPN.

Comparison interventions could be placebo, sham intervention, conventional pharmacological treatments, and any other management. In addition, patients who also received IPUPN in the control group will be excluded.

#### 2.3.4 Type of outcomes

The primary outcomes include IMs (such as C-reactive protein, white blood cell count, and body temperature), and incidence of new PUs (the proportion of participants developing any new PUs of any grade).

The secondary outcomes consist of time to ulcer development, quality of life as assessed by a validated tool, patient length of hospital stay (days or weeks), and adverse events.

### 2.4 Search strategy

We will carry out a rigorous literature search from Cochrane Library, MEDLINE, Embase, Cumulative Index to Nursing and Allied Health Literature, Allied and Complementary Medicine Database, and Chinese Biomedical Literature Database. We will search all those electronic databases from inception to the March 1, 2020 with no restrictions of language and publication status. The search strategy sample for Cochrane library is created in Table 1. We will also build similar search strategies for other electronic databases.

**Table 1:**
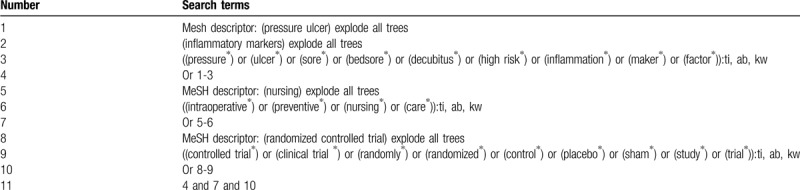
Search strategy utilized in Cochrane library.

In addition to the electronic databases, we will also examine clinical trial registries, dissertations, informal publication, and reference lists of all relevant reviews.

### 2.5 Data collection and management

#### 2.5.1 Study selection

All titles/abstracts of potential studies will be scanned by 2 independent investigators. After removing irrelevant studies, such as duplicates and nonclinical trials, full papers of the remaining trials will be read carefully against all inclusion criteria. Once any divergences occur between 2 investigators, a decision will be made through discussion or will be judged with the help of a 3rd investigator. Details of entire study selection will be summarized in a flowchart.

#### 2.5.2 Data collection

After study selection, data will be extracted based on the predefined standard data extraction form. Two investigators will independently extract the data from all included trials. Any disagreements between 2 investigators will be solved through discussion or consultation by a 3rd investigator. The extracted information includes study demographic information (such as 1st author, publication time, and country), patient characteristics (such as gender, age, and number of patients), study methods (such as study setting, randomization, and blind), interventions and comparators (such as types of therapies, dosage, and duration), outcomes (such as primary, secondary, and harm measurements), and other information (such as funding information).

#### 2.5.3 Missing data dealing with

If missing or insufficient information occurs, we will contact primary authors to obtain it by email or fax. We will analyze obtainable data if we cannot receive that information.

#### 2.5.4 Risk of bias assessment

Two investigators will appraise the risk of bias for each included trial using Cochrane risk of bias tool, which consists of 7 different items. Each one is judged as low risk of bias, unclear risk of bias, and high risk of bias. Any opposition between 2 investigators will be settled down by a 3rd investigator through discussion.

### 2.6 Data synthesis and analysis

We will use RevMan 5.3 Software to perform statistical analysis in this study.

#### 2.6.1 Measurement of treatment effect

For continuous data, it will be calculated using mean difference or standardized mean difference and 95% confidence intervals. For dichotomous data, it will be presented using risk ratio and 95% confidence intervals.

#### 2.6.2 Assessment of heterogeneity

Statistical heterogeneity among trial results will be identified using *I*^2^ index. *I*^2^ ≤ 50% will be regarded as not having statistical heterogeneity and a fixed-effects model will be exerted. On the contray, *I*^2^ > 50% will be considered as having statistical heterogeneity, and a random-effects model will be employed.

#### 2.6.3 Data synthesis

If *I*^2^ ≤ 50% and sufficient data are collected on the same outcome measurement, we will carry out a meta-analysis if necessary. Otherwise, if *I*^2^ > 50%, subgroup analysis will be conducted, and data will be synthesized more cautiously. If there is still substantial heterogeneity after subgroup analysis, the outcome data will not be suitable for pooling quantitative synthesis. Under such situation, we will report a narrative description with the information in the text to summarize and elaborate the characteristics and finding of individual trials.

#### 2.6.4 Subgroup analysis

Subgroup analysis will be carried out to explore the causes of significant heterogeneity based on the different study or patient characteristics, treatment and control types, and outcome measurements.

#### 2.6.5 Sensitivity analysis

When there are adequate trials, we will conduct sensitivity analysis to check the robustness and stability of conclusions by excluding low quality studies.

#### 2.6.6 Reporting bias

If adequate number of qualified trials is included, a funnel plot and Egger regression test will be performed to detect if there are any reporting biases.

### 2.7 Grading the quality of evidence

The quality of evidence and confidence for each outcome will be rated by 2 independent investigators according to the Grading of Recommendations Assessment, Development and Evaluation (GRADE) guidelines.^[28]^ Any differences between 2 investigators will be solved by a 3rd investigator through discussion.

## 3 Discussion

The PUs often occurs in patients with HRPU, especially when they are under long-term mobility or immobility, poor nutrition status, compromised blood flow, and neuropathy sensation. A variety of studies have reported that IPUPN can help to prevent HRPU and significantly affect IMs. However, no systematic review has conducted to explore the effects of IPUPN on IMs in patients with HRPU. Thus, it is very necessary to carry out this systematic review to examine the effects of IPUPN on IMs in patients with HRPU. The results of this study may provide informative evidence for both patients with HRPU and clinical practice.

## Author contributions

**Conceptualization:** Feng Jin, Hong-wei Liu.

**Data curation:** Feng Jin, Jing-ling Yan, Hong-wei Liu.

**Formal analysis:** Feng Jin, Yang Zhang, Jing-ling Yan, Kui-chen Zhou.

**Funding acquisition:** Hong-wei Liu.

**Investigation:** Hong-wei Liu.

**Methodology:** Feng Jin, Yan-jun Fu, Jing-ling Yan, Kui-chen Zhou.

**Project administration:** Hong-wei Liu.

**Resources:** Feng Jin, Yan-jun Fu, Yang Zhang, Jing-ling Yan, Kui-chen Zhou.

**Software:** Feng Jin, Yan-jun Fu, Yang Zhang, Jing-ling Yan, Kui-chen Zhou.

**Supervision:** Hong-wei Liu.

**Validation:** Yan-jun Fu, Yang Zhang, Hong-wei Liu.

**Visualization:** Feng Jin, Yan-jun Fu, Jing-ling Yan, Hong-wei Liu.

**Writing – original draft:** Feng Jin, Yan-jun Fu, Yang Zhang, Kui-chen Zhou, Hong-wei Liu.

**Writing – review & editing:** Feng Jin, Yang Zhang, Jing-ling Yan, Hong-wei Liu.

## References

[1] Gorecki C Brown JM Nelson EA Impact of pressure ulcers on quality of life in older patients: a systematic review. J Am Geriatr Soc 2009;57:1175–83. 19486198 10.1111/j.1532-5415.2009.02307.x

[2] Bennett G Dealey C Posnett J The cost of pressure ulcers in the UK. Age Ageing 2004;33:230–5. 15082426 10.1093/ageing/afh086

[3] Thomas DR Goode PS Tarquine PH Hospital-acquired pressure ulcers and risk of death. J Am Geriatr Soc 1996;44:1435–40. 8951312 10.1111/j.1532-5415.1996.tb04067.x

[4] Coleman S Nixon J Keen J A new pressure ulcer conceptual framework. J Adv Nurs 2014;70:2222–34. 24684197 10.1111/jan.12405 PMC4263098

[5] Loerakker S Manders E Strijkers GJ The effects of deformation, ischemia, and reperfusion on the development of muscle damage during prolonged loading. J Appl Physiol 2011;111:1168–77. 21757578 10.1152/japplphysiol.00389.2011

[6] Schols JM Heyman H Meijer EP Nutritional support in the treatment and prevention of pressure ulcers: an overview of studies with an arginine enriched oral nutritional supplement. J Tissue Viability 2009;18:72–9. 19427218 10.1016/j.jtv.2009.03.002

[7] Szewczyk MT Cwajda J Cierzniakowska K The guidelines for the effective prevention of pressure ulcers [in Polish]. Wiad Lek 2006;59:842–7. 17427502

[8] Brem H Nierman DM Nelson JE Pressure ulcers in the chronically critically ill patient. Crit Care Clin 2002;18:683–94. 12140919 10.1016/s0749-0704(02)00014-3

[9] Coleman S Gorecki C Nelson EA Patient risk factors for pressure ulcer development: systematic review. Int J Nurs Stud 2013;50:974–1003. 23375662 10.1016/j.ijnurstu.2012.11.019

[10] Hahnel E Lichterfeld A Blume-Peytavi U The epidemiology of skin conditions in the aged: a systematic review. J Tissue Viability 2017;26:20–8. 27161662 10.1016/j.jtv.2016.04.001

[11] Hampton S The use of alternating mattresses in the management and prevention of pressure ulcers in a community setting. Br J Community Nurs 2016;21: Suppl 9: S19–25. 27594310 10.12968/bjcn.2016.21.Sup9.S19

[12] Aizpitarte Pegenaute E García de Galdiano Fernández A Zugazagoitia Ciarrusta N Pressure ulcers in intensive care: assessment of risk and prevention measures [in Spanish]. Enferm Intensiva 2005;16:153–63. 16324543 10.1016/s1130-2399(05)73402-7

[13] Bours GJ De Laat E Halfens RJ Prevalence, risk factors and prevention of pressure ulcers in Dutch intensive care units. Results of a cross-sectional survey. Intensive Care Med 2001;27:1599–605. 11685300 10.1007/s001340101061

[14] Khlifi L Graiet H Sahli S Evidence of metabolic imbalance and oxidative stress among patients suffering from pressure ulcers. J Dermatolog Treat 2019;30:414–21. 30300033 10.1080/09546634.2018.1527991

[15] Ruh AC Frigo L Cavalcanti MFXB Laser photobiomodulation in pressure ulcer healing of human diabetic patients: gene expression analysis of inflammatory biochemical markers. Lasers Med Sci 2018;33:165–71. 29181642 10.1007/s10103-017-2384-6

[16] Krishnan S Vodovotz Y Karg PE Inflammatory mediators associated with pressure ulcer development in individuals with pneumonia after traumatic spinal cord injury: a pilot study. Arch Phys Med Rehabil 2017;98:1792–9. 28130082 10.1016/j.apmr.2016.12.018

[17] Reynolds TM Stokes A Russell L Assessment of a prognostic biochemical indicator of nutrition and inflammation for identification of pressure ulcer risk. J Clin Pathol 2006;59:308–10. 16505284 10.1136/jcp.2005.029405 PMC1860338

[18] Li L Zhai YH Bo QY Application of standardized operation procedures for intraoperative pressure ulcer management in high-risk patients with intraoperative pressure ulcer. Qilu Nurs J 2019;25:21–3.

[19] Zhang X Xu LX Application of intraoperative pressure ulcer care sheet in nursing of high-risk patients with pressure ulcer in operating room. Chin Foreign Women Health Res 2019;13:168–9.

[20] Chen JY The effect of intraoperative pressure ulcer nursing application on high-risk patients with surgical pressure ulcers. China Health Stand Manag 2019;10:152–4.

[21] Ouyang YY Chen CL Qian D Application effect of intraoperative pressure ulcer prevention and nursing measures on patients with high-risk pressure ulcers. J Clin Nurs 2018;17:53–5.

[22] Guan LL Analysis of the application effect of intraoperative pressure ulcer nursing sheet in the nursing of high risk patients with pressure ulcers in the operating room. Infect Int 2018;7:103–5.

[23] Zhu WJ Bai H Ding Y Research on the prevention effect of pressure ulcer risk quantitative assessment table on intraoperative pressure ulcers in patients with high-risk tumors of pressure ulcers. Contemp Nurs 2018;25:3–5.

[24] Huang L Application of intraoperative pressure ulcer nursing sheet in the nursing of high-risk patients with pressure ulcers in operating room. Capital Food Med 2018;25:93.

[25] Xie GQ Application of intraoperative pressure ulcer nursing sheet in high-risk patients with pressure ulcer in operating room. J Clin Nurs 2016;15:56–8.

[26] Pan FQ Application of intraoperative pressure ulcer nursing in high-risk patients with surgical pressure ulcers. J Pract Clin Med 2015;19:78–80.

[27] Moher D Shamseer L Clarke M Preferred reporting items for systematic review and meta-analysis protocols (PRISMA-P) 2015 statement. Syst Rev 2015;4:1. 25554246 10.1186/2046-4053-4-1 PMC4320440

[28] Guyatt GH Oxman AD Vist GE GRADE: an emerging consensus on rating quality of evidence and strength of recommendations. BMJ 2008;336:924–6. 18436948 10.1136/bmj.39489.470347.AD PMC2335261

